# Smart Parking Locks Based on Extended UNET-GWO-SVM Algorithm

**DOI:** 10.3390/s23208572

**Published:** 2023-10-19

**Authors:** Jianguo Shen, Yu Xia, Hao Ding, Wen Cabrel

**Affiliations:** College of Physics and Electronic Information Engineering, Zhejiang Normal University, Jinhua 321000, China; xiaxia@zjnu.edu.cn (Y.X.);

**Keywords:** automatic license plate recognition, parking locks, Raspberry Pi, gray wolf optimization, support vector machine, U-net

## Abstract

Due to the rapid increase in private car ownership in China, most cities face the problem of insufficient parking spaces, leading to frequent occurrences of parking space conflicts. There is a wide variety of parking locks available on the market. However, most of them lack advanced intelligence and cannot cater to the growing diverse needs of people. The present study attempts to devise a smart parking lock to tackle this issue. Specifically, the smart parking lock uses a Raspberry Pi as the core controller, senses the vehicle with an ultrasonic ranging module, and collects the license plate image with a camera. In addition, algorithms for license plate recognition based on traditional image-processing methods typically require a high pixel resolution, but their recognition accuracy is often low. Therefore, we propose a new algorithm called UNET-GWO-SVM to achieve higher accuracy in embedded systems. Moreover, we developed a WeChat mini program to control the smart parking lock. Field tests were conducted on campus to evaluate the performance of the parking locks. The test results show that the corresponding effective unlocking rate is 99.0% when the recognition error is less than two license plate characters. The average time consumption is controlled at about 2 s. It can meet real-time requirements.

## 1. Introduction

In recent years, the per capita car ownership in China has been increasing yearly, leading to a shortage of parking spaces in most cities. According to the data from the National Bureau of Statistics of China, in 2022, the total number of private cars reached over 320 million by the end of that year, representing a growth rate of 5.81% compared to the previous year. The existing parking spaces are mainly concentrated in office buildings, recreational areas, and residential areas. Among them, the management of parking spaces in residential areas is the most prominent issue, with frequent incidents of parking space occupation, which has caused significant difficulties in parking management and has compromised the legitimate parking rights of private car owners. On the other hand, parking spaces in residential areas during working hours and office building parking spaces at night are often left unused, resulting in the inefficient allocation of urban parking resources and difficulty in their effective utilization. A survey to address the above issues is provided below.

To effectively manage parking spaces, parking locks (also known as ground locks) have emerged. Common parking lock types are divided into two categories: mechanical manual locks and electronic remote-controlled locks (including remote controllers, mobile apps with Bluetooth, etc.). Traditional manual parking locks require vehicle owners to manually operate the lifting or lowering of the lock support to lock it. This process is both time-consuming and extremely inconvenient. Moreover, most manual locks do not have collision warning features, making them prone to damage and potentially causing damage to the vehicle. With the development of electronic technology, traditional mechanical parking locks have been further upgraded to electronic remote-controlled locks. Users can control the lifting and lowering of the parking lock through a remote controller or a smartphone with Bluetooth, significantly saving time compared to manual unlocking. Additionally, some electronic remote locks utilize sensors installed on vehicles to enable features such as proximity-based automatic unlocking, and they have received positive feedback. However, existing remote-controlled parking locks do not allow for the efficient sharing of parking spaces, resulting in low utilization rates of urban parking spaces.

Furthermore, most parking locks available on the market rely on close-range control by users to open or close them. Once the distance becomes too far, they may malfunction and fail to achieve the remote sharing of parking spaces. With technological advancements and an expanding market, smarter and more energy-efficient parking locks are becoming the trend of industry development. Therefore, designing a smart parking lock that combines the Internet of Things (IoT) and image recognition technology to automatically control the support bracket based on proximity sensing and the recognition of nearby license plates holds significant research value.

The present study attempts to devise an advanced smart parking lock to tackle this problem. Our approach centers around utilizing the Raspberry Pi 3B as the core controller, employing an ultrasonic distance module to detect vehicles, and incorporating a camera to collect license plate information. Traditional image-processing methods for license plate recognition often suffer from low accuracy and need high pixel resolution. To overcome these limitations, we have implemented a new algorithm known as UNET-GWO-SVM. Moreover, our system offers users the convenience of managing their parking spaces through a dedicated WeChat mini program, providing an intuitive and user-friendly interface.

Overall, this paper provides the following contributions:

1. A lightweight license plate localization model has been designed based on the U-Net architecture, which exhibits the precise localization of license plate regions even in complex scenes, demonstrating exceptional robustness.
2. Building upon a comprehensive analysis of license plate character recognition algorithms, we introduced the gray wolf optimization (GWO) algorithm into a character recognition model based on Support Vector Machines (SVMs). As a result, we constructed a GWO-SVM cascade recognition model designed explicitly for domestic license plate characters.
3. In conjunction with the aforementioned license plate recognition method, we propose a machine-vision-based smart parking lock. This parking lock system utilizes the Raspberry Pi development board as its central controller, ensuring seamless integration. Moreover, the lock is equipped with a 4G transmission module, establishing a connection with the IoT platform. This connectivity enables users to conveniently manage the lock via mobile terminals, facilitating efficient parking space sharing.

The rest of this article is organized as follows. In Section 2, we discuss the related work and the technology background. In Section 3, we describe the system architecture and explain the principle of operations. Section 4 shows the experimental results and discussions. Finally, the conclusion of this paper is given in Section 5.

## 2. Related Work

### 2.1. Review of Smart Parking Systems

In recent years, a number of studies have appeared on the security and management issues of smart parking [1]. Tang et al. combined IoT technology with traditional parking locks to design a real-time shared parking management system [2]. This solution provided a time-sharing approach to parking management. The aforementioned smart parking locks and their management solutions have expanded the functionality of parking locks to a certain extent. Xue et al. proposed an innovative bicycle intelligent lock [3]. The proposed method applied the multi-sensor fusion positioning algorithm to determine the location of the parking lot and the appropriateness of the positioning and realized the real-time monitoring and control of the system for bicycle parking through online monitoring and alarming software.

Later, many researchers applied geomagnetic detection technology to parking locks, further enhancing the sensitivity of the devices. Yao et al. designed a smart ground lock based on BLE and geomagnetic detection, improving the stability of the smart ground lock. Li et al. introduced geomagnetic detection technology into the parking detection module, creating a low-power Bluetooth parking lock. Lin et al. innovatively proposed a low-power remote-controlled parking lock powered by compressed air, effectively addressing issues such as the short battery life and the need for frequent battery replacement in conventional parking locks [4]. In recent years, driven by the sharing economy trend, research on smart-parking-lock-sharing technology has gradually increased. Chen et al. designed an embedded network-controllable parking lock that enabled remote management and parking-space-tracking functions. Tai et al. applied ultrasonic ranging technology and QR code technology [5] to design a self-sensing shared parking lock for private parking space sharing. Shi et al. proposed an induction-based wake-up control method, and experimental results demonstrated that the technology effectively reduced the power consumption and latency of the lock.

However, the aforementioned smart parking locks have limitations, such as a short remote control distance and the need for manual app operation, preventing them from achieving full automation. Subsequently, Liu et al. proposed a shared parking lock design combining microcontrollers with license plate recognition technology, further enhancing the intelligence of parking locks. However, there were issues with low recognition accuracy. Floris et al. designed a remote parking control system based on the IoT [6], enabling parking reservation, location navigation, and parking-space-leasing functions. Currently, the market share of [7] smart parking locks is still relatively small, and most locks require manual remote control by the vehicle owner or electronic tags, which causes inconvenience.

### 2.2. Overview of License Plate Recognition

Several researchers have proposed advanced automatic license plate recognition (ALPR) methods. He et al. developed a real-time and robust Chinese ALPR method [8] to accurately detect and identify inclined and twisted license plates in natural scenes. Pustokhina et al. presented a new OKM-CNN technique [9] that achieved a maximum overall accuracy of 0.981 on the Stanford Cars, FZU Cars, and HumAIn 2019 Challenge datasets. This technique effectively detects and recognizes license plates. Tourani et al. proposed a unified application for vehicle license plate detection and character recognition [10] that achieved high accuracy and real-time performance. The experimental results show that on 5719 images, the end-to-end accuracy reaches 95.05%, and the average time consumption is 119.73 ms. Zou et al. proposed a robust model for license plate recognition in complex environments using Xception, MobileNetV3, spatial attention mechanism, and Bi-LSTM methods. Experimental results show that the algorithm performs well without constraints, demonstrating the effectiveness and robustness of the model [11]. To make the model learn environment-independent and perspective-free semantic features effectively and efficiently, Zhang et al. put forward a shared adversarial training network with the prior knowledge of standard stencil-rendered license plates [12]. The author presented a one-stage ALPRNet [13] for multiple and mixed-style LP recognition, which equally treats LPs and characters as objects to detect and classify and conducts these two tasks simultaneously. 

Later, Henry et al. [14] developed a generalized method for multi-country license plate recognition based on the YOLOv3 network to address the limitation that automatic license plate recognition systems can only recognize license plates from a single country. Chen et al. [15] presented an end-to-end multi-branch attention neural network for simultaneous vehicle and license plate detection, which helps solve the problem that vehicle affects license plate detection when simultaneously detecting the vehicle and license plate. In addition, since the breakthrough of deep learning technology, related target detection algorithms have emerged in an endless stream, and the performance is outstanding, such as Faster R-CNN [16,17], SSD [18,19], or YOLO [20,21,22]. Based on the aforementioned technology, the recognition accuracy of the upgraded model is greatly improved. Besides license plate recognition algorithms, image quality is also an important issue [23]. A full-reference quality metric for light-field reconstruction, compression, and display quality evaluation was proposed to improve recognition accuracy [24,25].

## 3. The Proposed Smart Parking Lock System

### 3.1. System Architecture

The smart parking lock based on machine vision is an intelligent device that integrates license plate recognition technology with a vehicle lock. It aims to enhance safety and security by implementing an automatic lock function based on license plate recognition. The system uses machine vision algorithms to recognize the license plate numbers of approaching vehicles. By comparing the recognized license plate number with the pre-configured license plate number, the system determines whether to activate the automatic lock mechanism.

**Figure 1:**
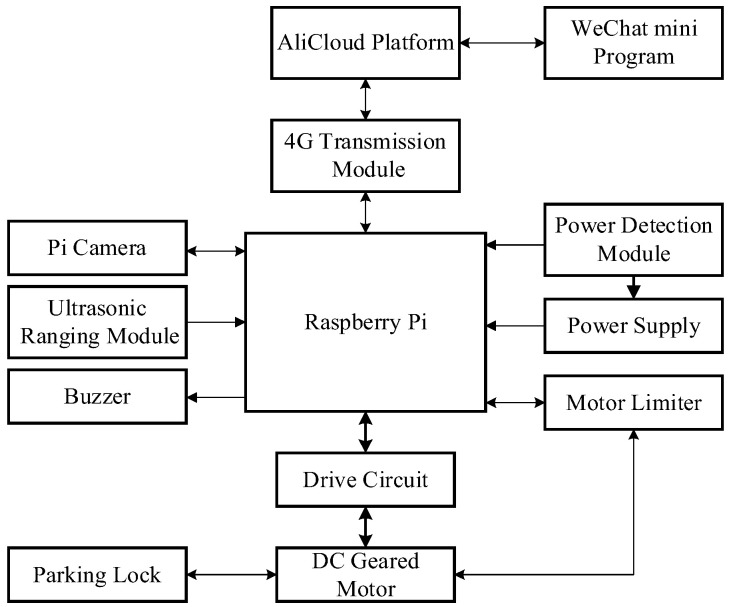
The system overview of the smart parking lock.

The operational mechanism of the smart parking lock, shown in Figure 1, can be described as follows. The user establishes a communication link with the embedded controller by means of a mobile terminal connected to the AliCloud platform. Subsequently, the associated license plate number is configured through the WeChat mini program, and vital information is transferred to the Raspberry Pi via the high-speed 4G mobile network. The controller incorporates the embedded, rapid, real-time license plate recognition algorithm proposed in this study. Once a vehicle is detected approaching the parking spot, the camera is automatically activated, triggering the license plate recognition algorithm to compare the captured license plate with the registered license plate number, thereby determining the unlock status of the vehicle lock. The parking lock also features additional functions, such as remaining battery power indication and occupied parking spot alert.

The Raspberry Pi serves as the core hardware of the smart parking lock and plays a crucial role in data processing and instruction transmission. The primary objective of the 4G transmission module is to establish IoT connectivity, enabling communication with the WeChat mini program and facilitating remote monitoring. The ultrasonic ranging module can detect nearby vehicles, while a camera on the Raspberry Pi captures the vehicle’s license plate, enabling image acquisition. The DC reduction motor controls the smart parking lock. Furthermore, the motor limiter is responsible for constraining the rotation angle and precise positioning of the DC motor. The buzzer serves to avert potential collisions with other vehicles.

### 3.2. License Plate Location

License plate location usually extracts the license plate area from the image that contains the license plate, according to the license plate features. At present, the standard license plate location algorithms can be divided into four categories: mathematical morphology [25], edge_ [26], color_ [27], and artificial neural networks [28,29,30]. In the traditional approach, mathematical morphological features, edge features, and color features are all prominent features of license plate positioning. However, due to the influence of the illumination and the tilt angle of the license plate, these methods cannot locate the license plate well in some complex scenes, such as those with blur and distortion. With the development of deep learning in recent years, the artificial neural network can effectively extract image features and carry out target tracking and semantic segmentation. License plate location is also a mode of image semantic segmentation, so this paper introduces the lightweight semantic segmentation network U-net for license plate location.

**Figure 2:**
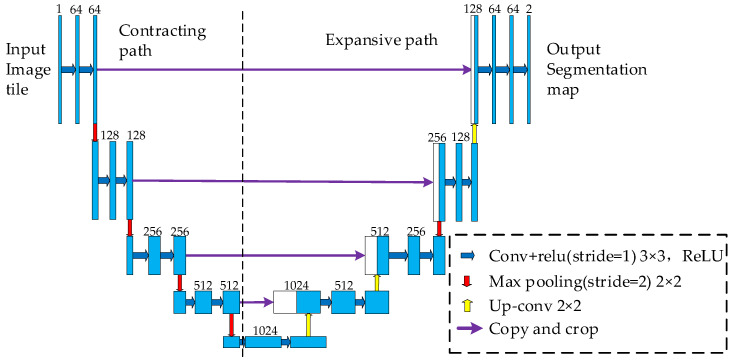
Diagram of network structure.

U-net was proposed [31] in the cell image segmentation contest in 2015. The structure of U-net is shown in Figure 2, with a ‘U’ shape. Its design can be divided into the contracting path and the expansive path. The contracting path is used for feature extraction at all levels of the image, and the expansive path uses up-sampling and other operations to fuse these features to locate the target area more accurately.

Compared to other convolutional neural networks, U-Net has several advantages in license plate localization, including its ability to capture contextual information, low sample requirements, end-to-end localization, and excellent image segmentation capabilities. These features make U-Net a powerful tool for processing license plate images and achieving accurate localization. Therefore, introducing the U-Net network into license plate localization is feasible in this section.

### 3.3. License Plate Character Recognition

After locating the license plate, we need to carry out the following operations: license plate rectangle correction, character segmentation, and character recognition. The license plate correction and character segmentation steps are usually relatively fixed. The character recognition algorithm significantly impacts the overall license plate recognition results, so this section focuses on license plate character recognition. Based on the requirements of low cost and high real-time performance in integration with the parking lock system, the traditional license plate character recognition method was selected in this study among various character recognition algorithms.

**Figure 3:**
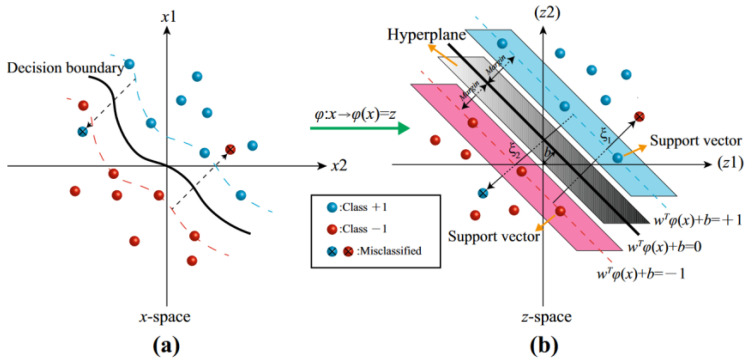
Graphical representation of SVM: (**a**) complex nonlinear classification problem representation in low-dimensional space and (**b**) linear classification problem representation in high-dimensional space.

Conventional methods can be divided into template matching, SVM, and the neural network. The template-matching algorithm extracts the preprocessed standard binary characters from the database and calculates the similarity between the target and the database template characters to classify characters. This method is simple in principle, poor in anti-interference, and significantly affected by environmental factors and the size of characters. The key to the SVM-based classifier is the decision boundary learned through the training process, which effectively separates the feature vectors of different character classes. This enables the SVM-based classifier to make classification judgments based on the position of the feature vectors relative to the decision boundary when recognizing new, unknown characters. The key to the SVM-based classifier is the decision boundary learned through the training process, which effectively separates the feature vectors of different character classes. This enables the SVM-based classifier to make classification judgments based on the position of the feature vectors relative to the decision boundary when recognizing new, unknown characters. Its graphical representation is shown in Figure 3.

The interval between the support vectors should be more significant to find the best hyperplane. As a result, the value of ω needs to be minimized. The solution of the optimal classification hyperplane can be divided into two cases according to whether the input data are linearly separable. The nonlinear classification problem can be converted into the following formula:

(1) $$R\left(\omega \right)=min\frac{1}{2}{\left|\left|\omega \right|\right|}^{2}+c\sum_{i=1}^{n}\left(\xi_{i}\right),s.t.\;\;y_{i}\left(\omega^{T}x_{i}+b\right)\geq 1-\xi_{i},\xi_{i}\geq 0,\;\;i=1,2,\ldots ,$$

The standard mathematical expressions of mapping Gaussian kernel function $K\left(x,y\right)$ are shown as follows:

(2) $$K\left(x,y\right)=e^{\left(-\frac{{\left||x-y|\right|}^{2}}{2\sigma^{2}}\right)}=e^{\left(-g{\left|\left|x-y\right|\right|}^{2}\right)}$$

where σ is the standard deviation of the Gaussian function distribution. After a series of mathematical derivations, the hyperplane classification decision function is as follows:

(3) $$F_{k}\left(x\right)=sgn\left(\sum_{j=1}^{N}{\alpha_{i}}^{T}y_{i}K\left(x_{i},x_{j}\right)+b\right)=sgn\left(\sum_{j=1}^{N}{\alpha_{i}}^{T}y_{i}e^{\left(-g{\left|\left|x_{i}-x_{j}\right|\right|}^{2}+b\right)}\right),\;s.t.\;\;c\geq \alpha_{i}\geq 0$$

where c is the penalty factor, and g is the kernel parameter, which significantly influences the classification effect of SVM. At the same time, the traditional algorithm generally uses the practical value as its parameter value. Specifically, the value of c represents the degree of penalty in SVM. A large value can lead to overfitting, where the model becomes too complex and may fit the training data too closely. On the other hand, a small value can result in underfitting, where the model is too simple and fails to capture the underlying patterns in the data. The kernel parameter g represents the clustering degree of Gaussian kernel mapping. If g is too large, distinguishing the data after mapping will not be easy. If g is too small, the classification will be too fine, resulting in overfitting.

Therefore, we use various swarm intelligence optimization algorithms to find the best parameter combination of the SVM classifier to improve the Chinese character recognition accuracy. Compared with various swarm optimization algorithms, the GWO algorithm was finally selected to optimize the kernel parameter g and penalty factor c of SVM.

**Figure 4:**
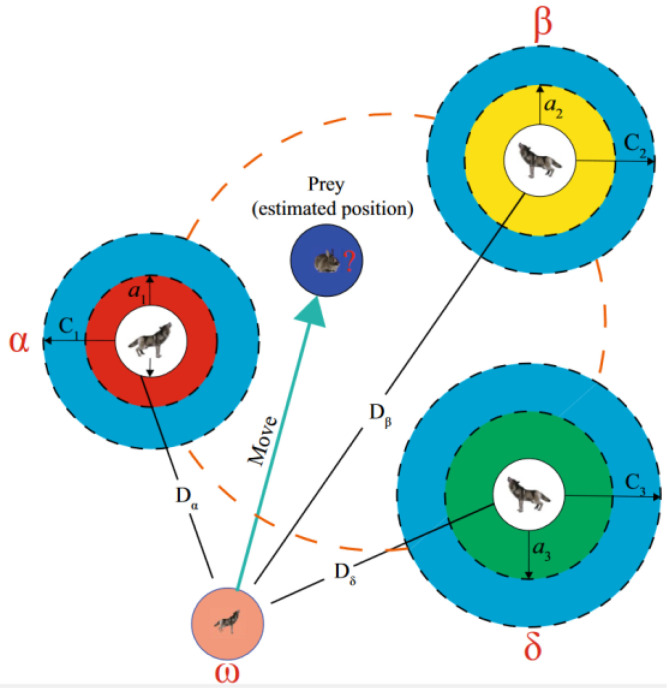
GWO algorithm diagram.

The GWO algorithm is a strategy to search for the optimal target by artificially imitating the hunting mechanism of a gray wolf population to surround, hunt, and attack prey. Among them, α, β, and δ are divided according to the social hierarchy of wolves, which are expressed as superior, sub-optimal, and third-optimal solutions, respectively, and their positions in the search space represent the optimal solution. The remaining individual ω will update its search position according to the relative position and finally search for prey. The specific search process is visualized in Figure 4.

In this study, the widely used Histogram of Oriented Gradients (HOG) was chosen as the feature extraction method for license plate characters [32]. The GWO-SVM classifier is trained to recognize 7-bit license plate characters. In addition, since the stroke structure of Chinese characters is more complex than numbers and letters, a Chinese character sub-classifier is separately trained for 31 provinces in the mainland, and the final license plate character classifier is a cascade of letter and number sub-classifiers. The GWO-SVM model approximates the actual value within the maximum number of iterations by conducting continuous optimization tests. The specific process can be summarized in two steps:

Step 1. The best parameter values (c, g) are used in the search parameter interval of the gray wolf population. The two-dimensional position vector of the gray wolf is set in the search space according to the number of optimization parameters to characterize the target parameter. And the wolf pack position is updated through iteration. Based on the individual functions of the first three wolves, the target position vector is solved approximately.

Step 2. The target position vector solved in the previous step is assigned to the SVM model as the (c, g) parameter. Thus, the SVM model is retrained, and the test set is predicted. An appropriate fitness function is set to characterize the training result, and the wolf pack is traversed to calculate the individual fitness value. The three new leader wolves of the wolf pack are determined through the maximum fitness value, and the gray wolf model parameters are updated.

The SVM parameter values are optimized after continuous loop iteration of the above two steps. Finally, a recognition model suitable for the training dataset is constructed.

### 3.4. Combination Algorithm

This study uses U-net to locate the license plate area. Then, the traditional horizontal and vertical projection analysis is used to extract and segment characters. This method uses the pixel brightness difference between the license plate characters. It determines the segmentation threshold by analyzing the peaks and troughs of the gray-scale image’s horizontal projection and vertical projection histogram to segment the license plate characters. Finally, the SVM model with optimized parameters can recognize the license plate number.

### 3.5. System Software Design

The system software design includes the Raspberry Pi control program, the WeChat mini program, and the IoT access platform. The Raspberry Pi control program detects vehicles automatically, captures license plates, recognizes the license plates, accesses the IoT, and sends instructions and other functions. The WeChat mini program allows users to manage parking locks anytime and anywhere. As the data transfer station between the Raspberry Pi and the WeChat mini program, the IoT access platform establishes the communication connection between them. In addition, the system uses the MQTT communication protocol.

The workflow of the smart parking lock system can be summarized as follows:

(1) Users can log in to the management page through the mobile client (the proposed method uses the WeChat mini program as the management software) and bind the license plate number.
(2) When the vehicle is close to the parking lock, the ultrasonic ranging module of the parking lock calls the CSI camera to capture the license plate according to the set threshold.
(3) The Raspberry Pi is utilized to process the collected image data and accurately extract the license plate number as the output.
(4) The Raspberry Pi controller sequentially compares the recognition result with the bound license plate numbers. If a match is found, the motor is driven to unlock. If there is no match, the alarm is triggered.

**Figure 5:**
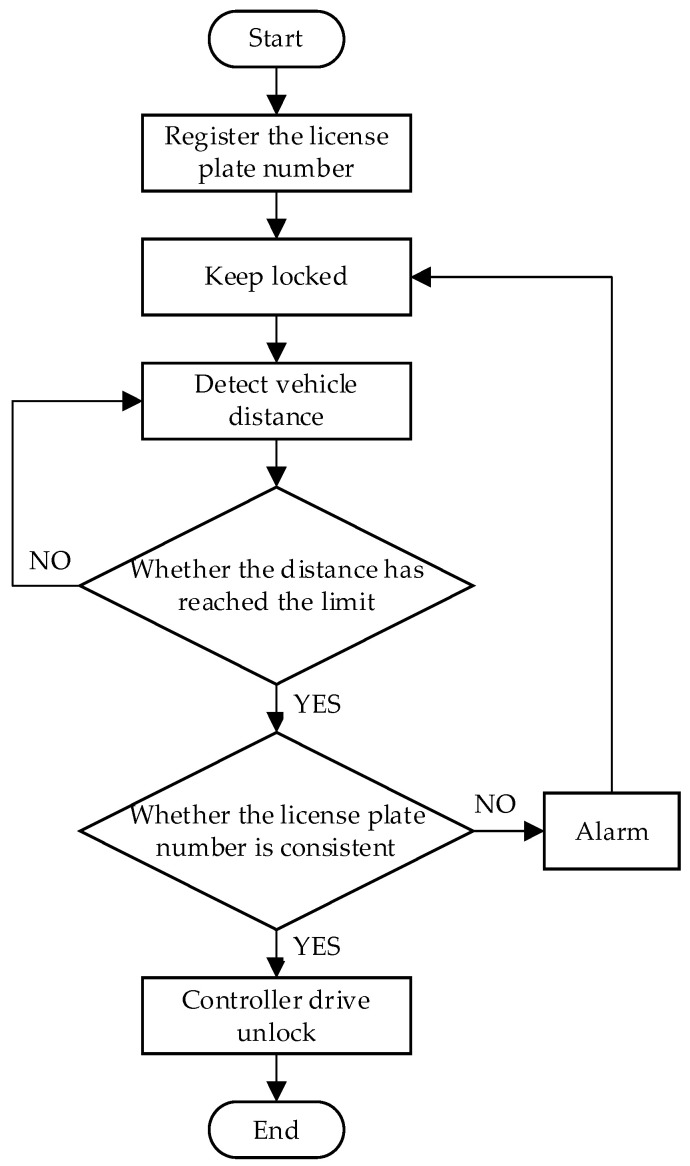
The specific flowchart.

The specific flowchart is shown in Figure 5.

## 4. Experimental Results and Analysis

### 4.1. License Plate Location Based on U-Net

**Figure 6:**
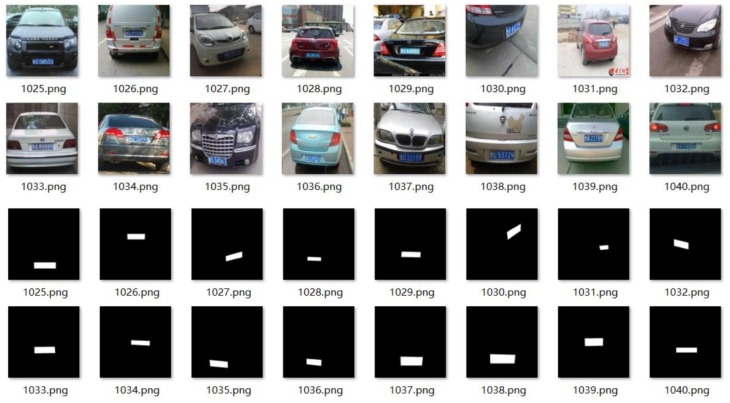
Dataset annotation diagram.

Due to U-Net’s robustness with smaller training datasets, this study used a dataset of 1300 high-definition blue-and-white license plates as the experimental dataset. The training-to-validation dataset ratio was 4:1. As shown in Figure 6, the training dataset was manually labeled, with the original images used as sample data and the annotated binary images used as labels. The license plates are segmented on the original images during license plate localization.

**Figure 7:**
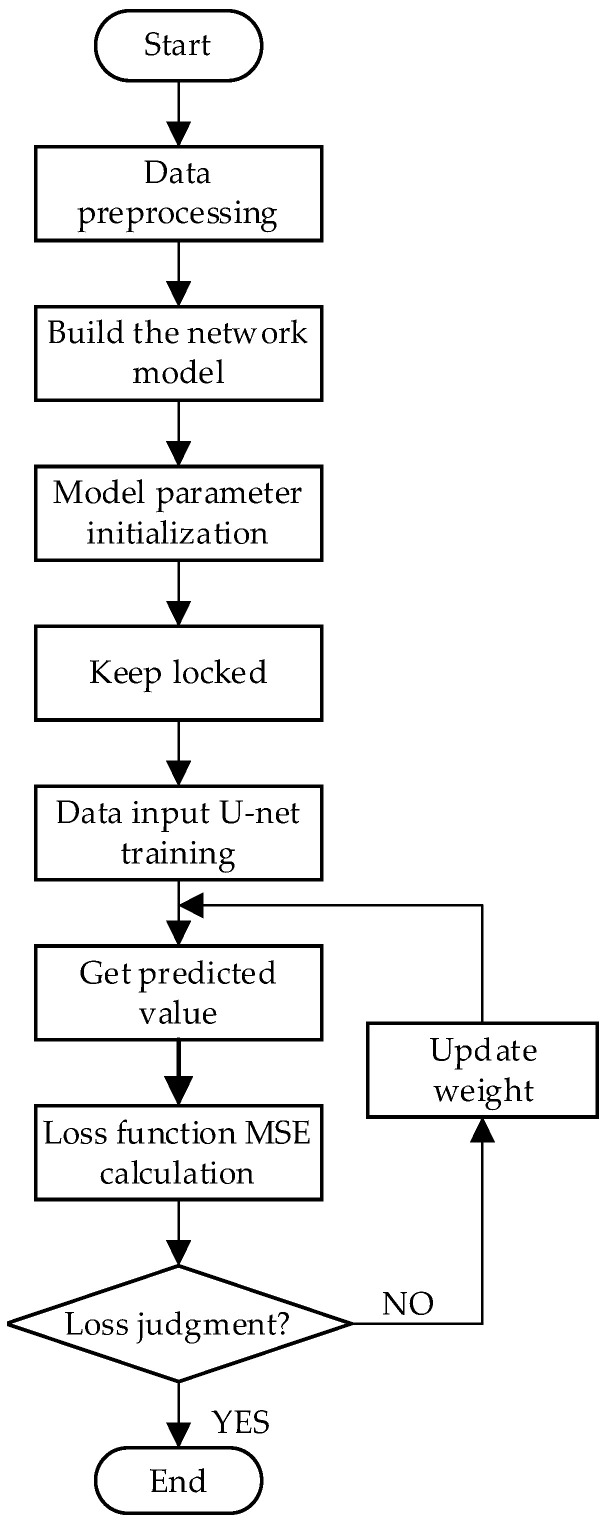
Training flowchart.

The computer vision library OpenCV was leveraged to ingest the pre-labeled dataset into U-net while meticulously configuring the requisite model parameters in adherence to the conventional training protocol of deep learning artificial neural networks. Subsequently, repetitive training was conducted based on evaluation metrics and the loss function until convergence was achieved. The network model training flowchart is shown in Figure 7.

**Figure 8:**
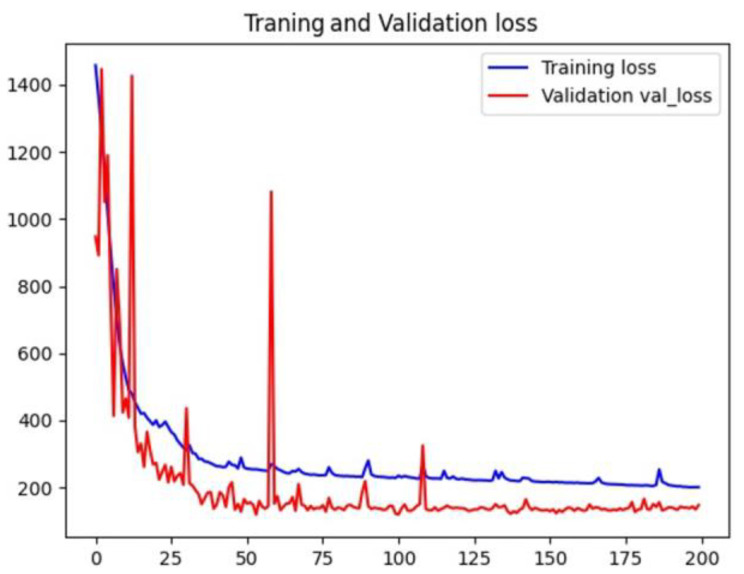
Training loss curve.

The widely used Adam optimizer was used. The default learning rate is 0.001. We used the license plate localization accuracy as an evaluation metric, while the mean square error (MSE) was used as a loss function. The loss value during training is shown in Figure 8.

**Figure 9:**
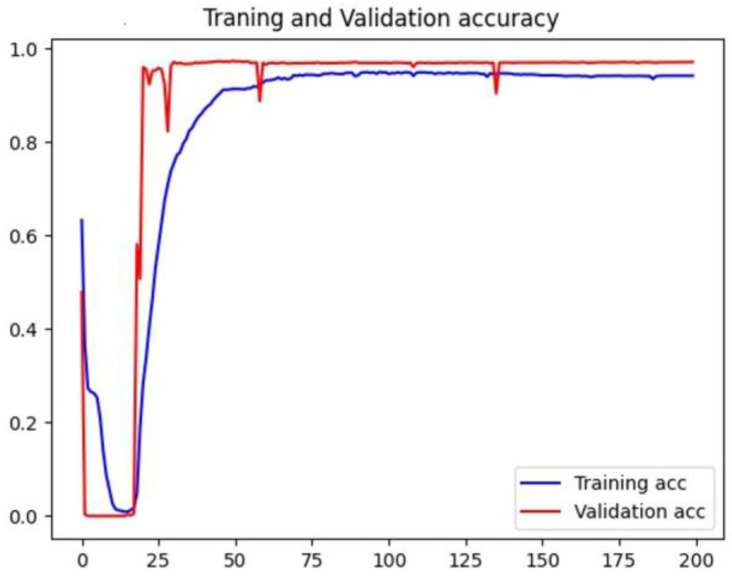
Accuracy rate change curve.

Moreover, the trend of the accuracy rate of the training set and the validation set is shown in Figure 9.

During the training process, the loss function value of the training set showed a downward trend. In contrast, the loss function value of the verification set initially significantly oscillated and then gradually decreased as the iteration progressed, and it converged from the 75th epoch. The specific loss value gradually stabilized around the 220th epoch, and the verification set loss value stabilized at the 150th epoch. During training, the accuracy of the model training set declined before the 16th epoch and then gradually increased and converged at the 75th epoch to 0.95. After that, the curve stabilized, and the accuracy of the model validation set was zero before the 16th epoch. As the accuracy of the training set increased, it gradually rose and stabilized at 0.97. In summary, the training of U-Net achieves the expected effect.

### 4.2. Character Recognition Based on SVM

The license plates used in the Chinese mainland consist of 31 Chinese characters, 10 Arabic numbers, and 24 uppercase English letters, excluding the characters ‘O’ and ‘I’, based on their arrangement on the license plate.

In the character recognition stage, the standard character training set employs segmented binary characters as the training set for the SVM model. This helps reduce the computational burden for character training and enhances the distinctive features of the characters. This study utilized a dataset comprising 18,289 images with a resolution of 28 × 28 pixels. Among these, 13,177 images were selected as the training set, while 5112 images were designated as the test set. The maximum iteration number was set to 100, and the number of gray wolves was set to 20. The optimization boundary range was between 0.01 and 100 based on the reference values of the traditional penalty factor ‘c’ and the kernel parameter ‘g’. Subsequently, the GWO-SVM model was trained using the parameter values of the GWO algorithm, and the resulting experimental data were analyzed.

This paper presents a comparative analysis of various metaheuristic algorithms, namely, the genetic algorithm, particle swarm algorithm, firefly algorithm, cuckoo algorithm, and gray wolf algorithm. The experimental results are presented in Table 1.

The test result conclusively demonstrates that GWO-SVM yields the highest accuracy in character recognition. Due to the fact that the recognition accuracies of uppercase letters and Arabic numerals are close to each other, we trained the Chinese and English classifiers separately. Then, they were cascaded into the license plate character recognizer, which helps to improve the overall parameter optimization classification effect. In addition, the GWO-SVM model has a more powerful global optimization capability and higher recognition accuracy than the above optimization algorithms.

In addition, the optimization speed of the proposed method was tested on 350 samples. The specific experimental data are shown in Table 2.

The results indicate that the GWO-SVM model has the fastest iteration time and optimization speed.

### 4.3. License Plate Recognition Experiment

**Figure 10:**
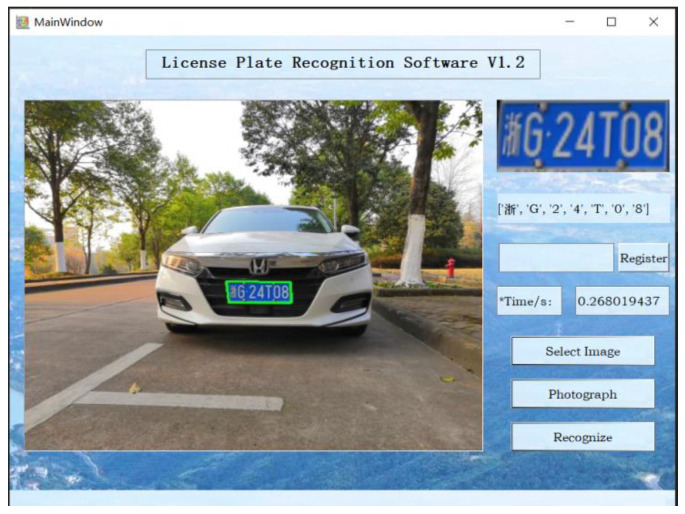
The user software graphical user interface.

Based on the proposed method, we designed a lightweight license plate recognition model suitable for embedding. Moreover, the user software graphical user interface based on the license plate recognition algorithm is shown in Figure 10.

Figure 10 shows that the single-frame time for license plate identification running on a CPU is about 300 ms. The recognition of license plate images with excessive tilt angles takes only 300 ms, which meets the real-time requirements.

In addition, we also carried out an algorithm comparison experiment and selected a set of 175 challenging license plate pictures as the test dataset. The images in the dataset have some problems, such as tilt, license plate distortion, and character blur, and the size and resolution of the pictures are different, which significantly increases the difficulty of license plate recognition. The experiments were run in the TensorFlow environment of the computer. The investigation involved the UNET-GWO-SVM algorithm, traditional image-processing recognition algorithm, and Chinese open-source neural network hyper-LPR model. The specific test data are shown in Table 3.

The comparative test of the above algorithms shows that the license plate recognition algorithm based on image processing has low recognition accuracy for challenging test samples, which is above 70.0%. However, the recognition speed is fast, and the average single-frame time is 50 ms~70 ms. The license plate recognition algorithm based on the neural network has high accuracy. However, the average recognition time is also longer. The experimental results show that the hyper-LPR algorithm, a domestic high-performance, open-source algorithm, has high recognition accuracy for a license plate with a slight inclination angle. However, it also consumes many resources and takes a long time. Compared with the hyper-LPR model, the UNET-GWO-SVM model designed in this paper dramatically improves the positioning accuracy and recognition speed. On the other hand, there is still a gap compared with different end-to-end neural network recognition algorithms [41,42,43,44]. However, they consider the applicability of embedded hardware. The license plate recognition algorithm designed in this paper still has excellent application prospects and can fully meet the functional requirements of a smart parking lock.

**Figure 11:**
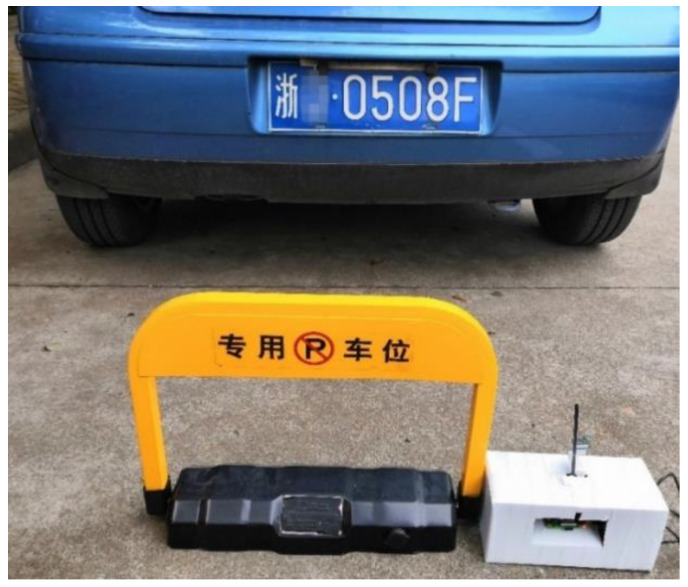
Parking lock effect diagram.

Finally, we integrated the above algorithm into a parking lock to complete a smart parking lock. The parking lock equipment comprises a mechanical device and a control center. The automatic device includes a modified motor drive module, drive power supply, bracket lock, and drive motor. The control center encapsulates the extended Raspberry Pi controller [45]. The parking lock device automatically detects and recognizes the license plate number through the Raspberry Pi controller and sends instructions to the mechanical device to control the opening and closing of the ground lock. A physical picture of the hardware device package is shown in Figure 11.

Based on the device, this study carried out a function test for 102 blue cars in the campus parking lot and collected the relevant data on license plate recognition and the effective opening of the parking lock. Table 4 shows the results of the experiments.

The results show that the system can correctly recognize 96 blue brand vehicles in the recognition test of 102 blue brand vehicles on campus. When the recognition error is less than two license plate characters, the number of cars recognized by the system even reaches 101. The corresponding effective unlocking rate is 99.0%, and the average time consumption is controlled at about 2 s, which can meet the real-time requirements.

On the one hand, considering the limitations of the character segmentation and character recognition algorithms, the error rate of single-character recognition, especially Chinese character recognition, is high. On the other hand, this system can pass the license plate determination if it is similar to the license plate number used as a template for string comparison. However, in reality, the possibility of nontarget license plate numbers and binding license plate numbers being similar in charge is very low. Therefore, this paper takes the similarity of license plate characters as the basis of unlocking, and unlocking occurs when it reaches a 70% matching degree to improve parking lock unlocking efficiency.

## 5. Conclusions

This paper mainly focuses on two aspects: the design of an efficient license plate recognition algorithm for lightweight embedded hardware and the implementation of smart parking locks. Based on the study of existing license plate recognition algorithms and combined with the application requirements of parking locks, a medical image segmentation network U-net is introduced to improve the license plate positioning accuracy; the gray wolf algorithm (GWO) is introduced to optimize the SVM model, and a GWO-SVM-based character recognition model is established to improve the license plate character recognition accuracy with balanced resources and performance. Combining the above positioning and character recognition algorithms, a UNET-GWO-SVM license plate recognition algorithm that can be applied to embedded systems is proposed, and a smart parking lock based on machine vision is designed and implemented in combination with the mechanics of traditional parking locks.

The main research work of this paper can be summarized as follows:

(1) Given the limitations of embedded hardware performance, this study aims to improve existing license plate recognition algorithms. Specifically, to address the issue of inaccurate license plate localization in complex scenarios, the U-net network is introduced for image segmentation, leading to the development of a lightweight localization model. Comparative tests were conducted between this localization model and traditional localization algorithms to demonstrate the effectiveness of the proposed approach.

(2) To address the issue of using empirical values and the lack of targeted optimization for the parameters in the traditional SVM model, the GWO (gray wolf optimizer) algorithm is introduced to optimize the penalty factor and kernel parameters. As a result, the GWO-SVM recognition model is established. The GWO-SVM model exhibits an average improvement of over 5% in recognition accuracy compared to the original model. Additionally, the GWO-SVM model demonstrates fast convergence during iterations and achieves high accuracy in character recognition.

(3) A smart parking space lock based on machine vision was designed using a Raspberry Pi development board as the core controller. The parking space lock can automatically detect the vehicle driving in and collect the license plate information, and then it calls the UNET-GWO-SVM model for license plate recognition and automatically controls the ground lock. The test results show that the complete recognition success rate reaches 97% in a campus parking lot, and the average time is about 2 s, which meets actual needs.

## Figures and Tables

**Table 1:** Comparison results of character recognition experiments.

Algorithms	Chinese Character	Letter	Arabic Numerals
SVM [33]	87.09%	92.00%	95.00%
GA-SVM [34]	96.88%	99.20%	99.14%
PSO-SVM [35]	95.14%	99.42%	99.43%
FA-SVM [36]	96.16%	99.31%	99.35%
CS-SVM [37]	96.06%	98.91%	99.09%
GWO-SVM [38]	97.97%	99.77%	99.80%

**Table 2:** Comparison results of model optimization speed.

Algorithms	The Time for Optimization of Small Samples (s)	Parameter Settings
GA-SVM [34]	613.04	Dataset: 350 Iterations: 20 Population: 10
PSO-SVM [35]	243.16	
CS-SVM [36]	106.46	
FA-SVM [37]	70.10	
GWO-SVM [38]	65.37	

**Table 3:** Test results of license plate recognition algorithm.

Algorithms	Recognition Accuracy (%)	Average Time (s)
Traditional image processing [39]	72.0	0.053
Hyper-LPR [40]	83.4	1.204
UNET-GWO-SVM	94.3	0.216
UNET+CNN (AlexNet)	93.7	0.923

**Table 4:** Field measurement datasheet of the parking lock.

Algorithms	Experimental Parameters	Error = 0	Error ≤ 1	Error ≤ 2
UNET-GWO- SVM	Identification quantity	96	100	101
	Effective unlocking rate (%)	94.1%	98.0%	99.0%
	Average time (s)	2.681		

## Data Availability

Not applicable.
